# A case of severe acute necrotizing pancreatitis in a 38-year-old woman postpartum due to a parathyroid adenoma

**DOI:** 10.3205/iprs000115

**Published:** 2017-08-21

**Authors:** Holger Rupprecht, Julia Reinfelder, Alp Turkoglu

**Affiliations:** 1 Department of Visceral, Thoracic, and Vascular Surgery, Surgical Clinic Fürth, Germany

**Keywords:** pregnancy, hyperparathyroidism, parathyroid adenoma, pancreatitis

## Abstract

Lethal necrotizing pancreatitis postpartum due to primary hyperparathyroidism caused by a parathyroid adenoma can be considered as a rarity. Due to the unspecific clinical signs and uncommonness this disorder may be overseen very easily. The reported case illustrates the very importance of early diagnosis of this endocrine disorder in pregnancy in order to avoid a lethal course.

## Zusammenfassung

Eine tödlich verlaufende postpartale nekrotisierende Pankreatitis durch einen primären Hyperparathyreoidismus bei einem Nebenschilddrüsenadenom ist eine absolute Rarität. Wegen der unspezifischen Symptome und der Seltenheit der Erkrankung wird diese leicht fehlinterpretiert. Dieser Fall demonstriert die enorme Bedeutung einer frühzeitigen Diagnose dieser endokrinen Störung während der Schwangerschaft, um einen tödlichen Verlauf zu verhindern.

## Introduction

Primary hyperparathyroidism is a common endocrine disorder. The vast majority of the cases are due to a single adenoma, followed by hyperplasia (15–20%) and parathyroid cancer (1%) [3]. This disorder is rarely diagnosed during pregnancy and it is the most common cause of hypercalcemia in pregnancy. It may be associated with severe complications (like in our case) and significant maternal and fetal morbidity.

## Case description

**Figure 1:**
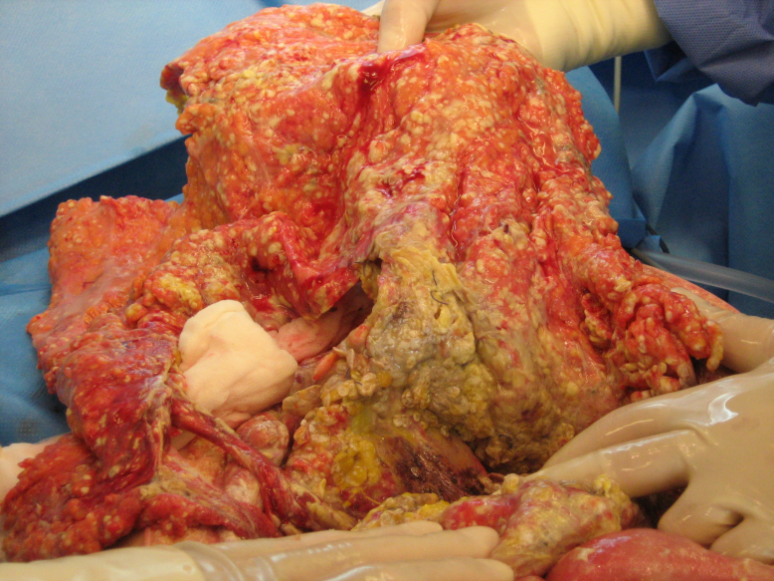
Intraoperative photographic documentation: The whole greater omentum covered with calcifications and the necrotic areas.

**Figure 2:**
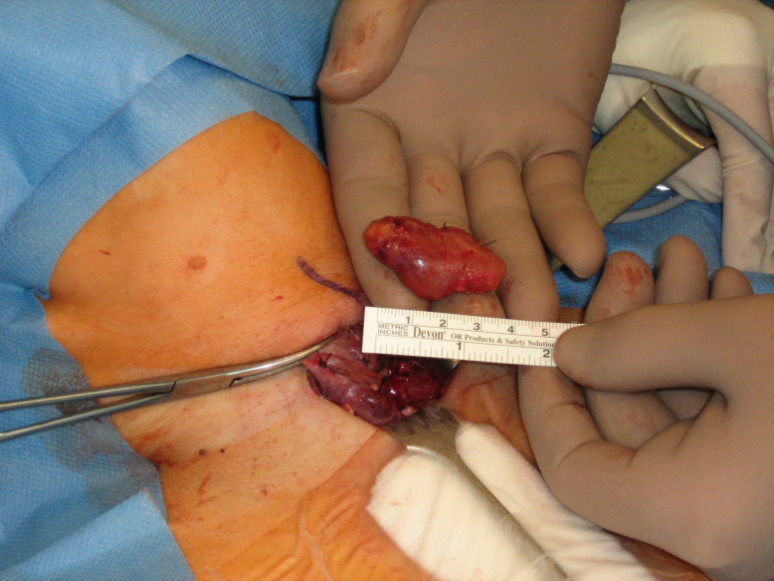
Intraoperative photographic documentation: Exploration of the neck. A 4.5 g weighing adenoma of the lower left parathyroid gland.

A 38-year-old woman presented in the gynecological emergency unit complaining about massive upper abdominal pain and vomitus. The pregnancy to date of admission developed without any pathological findings in preventive medical examinations. When premature contractions in cardiotocography were seen, a primary tocolysis was started. Results of blood tests and abdominal ultrasonography lead to the strong suspicion of a severe exsudative pancreatitis (Table 1 (Tab. 1)). The patient was transferred to the intensive care unit immediately. Due to a silent cardiotocogram during following surveillance an emergency caesarean section was performed in spinal anesthesia. In the following hours the clinical situation aggravated rapidly. The performed computer tomography showed a massive necrotizing pancreatitis. The further decay of vital signs (systolic blood pressure 70 mmHg) and development of a fulminant abdominal compartment syndrome (bladder pressure higher than 40 mmHg) necessitated emergency laparotomy. Intraoperative about 12 liters of hemorrhagic ascites could be aspirated, the whole greater omentum was covered with calcifications and the necrotic areas expanded down to the lesser pelvis (Figure 1 (Fig. 1)). We performed a necrosectomy with the evacuation of the tissular debris and the abscesses followed by a vacuum assisted closure (VAC) of the abdomen. Subsequently, the patient’s vital signs could only be stabilized with high doses of catecholamines. Additionally, haemofiltration was necessary to control an increasing acidotic metabolism as well as the hypercalcaemia. As hypercalcaemia is an unusual finding in pancreatitis we performed an ultrasound of the neck and found an adenoma of the left lower parathyroid gland. The strong suspicion of a primary hyperparathyroidism was confirmed with parathormone measurement which showed a massively increased level of 1,330 pg/ml (normal range 15–65 pg/ml). As a result of these findings we performed a relaparotomy postoperative day one. Intraoperative we observed a rapid progression of the necrosis and the whole intestine showed signs of a disturbed perfusion. After reapplication of the VAC system the neck was explored and a 4.5 g weighing adenoma of the lower left parathyroid gland was removed (Figure 2 (Fig. 2)). In the following days the VAC system was changed every two days during maximum intensive care. Two weeks after primary surgery a subtotal colectomy, resection of the left pancreas, and splenectomy were necessary. After 2 more days the maximum intensive care was terminated due to complete gangrene of the intestine, liver necrosis, and uncontrollable candida sepsis. After 6 months the child was normally developed and healthy.

## Discussion

Lethal necrotizing pancreatitis postpartum due to primary hyperparathyroidism caused by a parathyroid adenoma can be considered as a rarity. It is associated with a significant increase in morbidity and mortality of mother and child [3], [4], [8], [9], [11], [12]. Due to the uncommonness of this metabolic disorder and its unspecific clinical signs, hyperparathyroidism may be overseen and its symptoms disregarded as pregnancy associated [5], [10]. Additionally, the serum calcium level is not a routine parameter determined in pregnancy. As a conclusion, we suggest serum calcium determination once in each trimester of pregnancy. If hypercalcaemia is detected, the parathormone level must be measured and an ultrasonography of the neck should be performed. In case of suspicious findings in these examinations, an MRI of the neck and the mediastinum is indicated to investigate the possibility of multiple or ectopic adenomas [6], [11], [15]. As a matter of course computed tomography and scintigraphy are contraindicated due to potential fetal impairment [10], [15], [17]. 

As a diagnostic investigation in case of acute abdominal pain in pregnancy an abdominal ultrasound should be performed. If there are signs of pancreatitis an MRI is required for clarification. Additionally in case of elevated serum calcium a measurement of the parathormone level and an MRI of the neck and mediastinum are indispensable [1], [2], [11], [15], [17]. Increased calcium level in combination with pancreatitis is suspicious of hyperparathyroidism as in most cases calcium level decreases in case of pancreatitis [11], [14]. 

In case of primary hyperparathyroidism without pancreatitis the adenoma should be surgically removed during gestation preferably in the second trimester. If pancreatitis occurs the parathyroid adenoma must be removed immediately. Parathyroidectomy carries a very low rate of complication even in urgent surgery [4], [5], [9], [13], [16], [17]. In contrast, it is unreasonably dangerous to wait until accouchement. In our case the complicated and lethal progress is due to the delayed diagnosis and adenoma resection [7], [8].

## Notes

### Competing interests

The authors declare that they have no competing interests.

## Figures and Tables

**Table 1:**
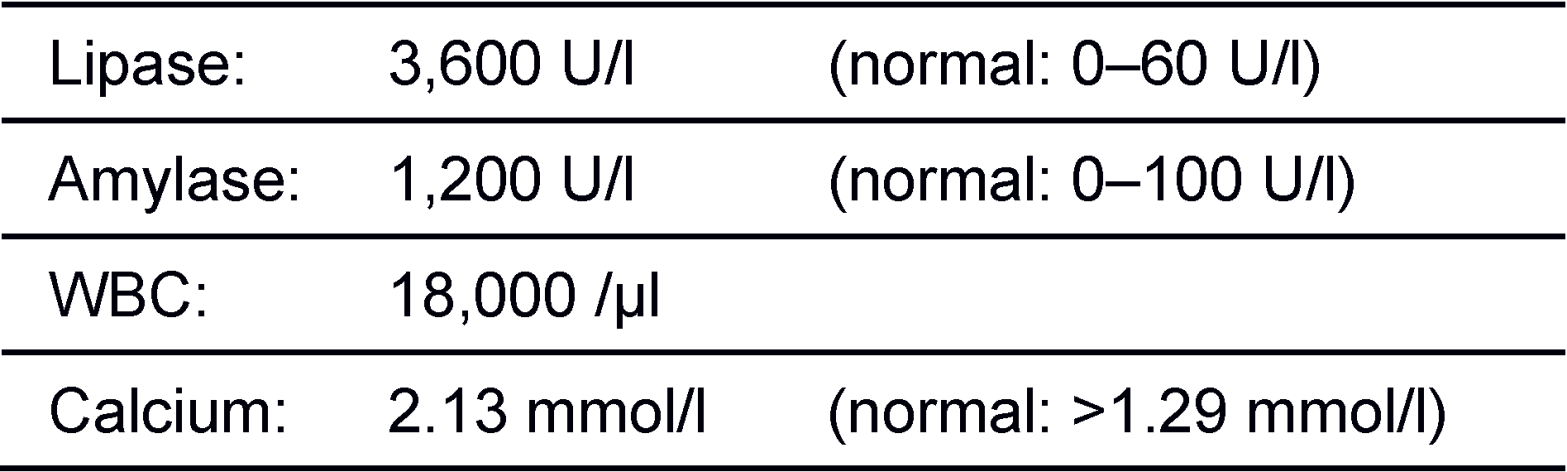
Laboratory results at admission

## References

[1] Biondi A, Persiani R, Marchese M, Cananzi F, D'Ugo D (2011). Acute pancreatitis associated with primary hyperparathyroidism. Updates Surg.

[2] Carella MJ, Gossain VV (1992). Hyperparathyroidism and pregnancy: case report and review. J Gen Intern Med.

[3] Diaz-Soto G, Linglart A, Sénat MV, Kamenicky P, Chanson P (2013). Primary hyperparathyroidism in pregnancy. Endocrine.

[4] Eddy JJ, Gideonsen MD, Song JY, Grobman WA, O'Halloran P (2008). Pancreatitis in pregnancy. Obstet Gynecol.

[5] Higgins RV, Hisley JC (1988). Primary hyperparathyroidism in pregnancy. A report of two cases. J Reprod Med.

[6] Imachi H, Murao K, Kontani K, Yokomise H, Miyai Y, Yamamoto Y, Kushida Y, Haba R, Ishida T (2009). Ectopic mediastinal parathyroid adenoma: a cause of acute pancreatitis. Endocrine.

[7] Kelly TR (1991). Primary hyperparathyroidism during pregnancy. Surgery.

[8] Kondo Y, Nagai H, Kasahara K, Kanazawa K (1998). Primary hyperparathyroidism and acute pancreatitis during pregnancy. Report of a case and a review of the English and Japanese literature. Int J Pancreatol.

[9] Kort KC, Schiller HJ, Numann PJ (1999). Hyperparathyroidism and pregnancy. Am J Surg.

[10] Krysiak R, Wilk M, Okopien B (2011). Recurrent pancreatitis induced by hyperparathyroidism in pregnancy. Arch Gynecol Obstet.

[11] Lenz JI, Jacobs JM, Op de Beeck B, Huyghe IA, Pelckmans PA, Moreels TG (2010). Acute necrotizing pancreatitis as first manifestation of primary hyperparathyroidism. World J Gastroenterol.

[12] Mestman JH (1998). Parathyroid disorders of pregnancy. Semin Perinatol.

[13] Pitchumoni CS, Yegneswaran B (2009). Acute pancreatitis in pregnancy. World J Gastroenterol.

[14] Ranson JH (1982). Etiological and prognostic factors in human acute pancreatitis: a review. Am J Gastroenterol.

[15] Rooney DP, Traub AI, Russell CF, Hadden DR (1998). Cure of hyperparathyroidism in pregnancy by sternotomy and removal of a mediastinal parathyroid adenoma. Postgrad Med J.

[16] Schnatz PF, Curry SL (2002). Primary hyperparathyroidism in pregnancy: evidence-based management. Obstet Gynecol Surv.

[17] Wilson RD, Martin T, Christensen R, Yee AH, Reynolds C (1983). Hyperparathyroidism in pregnancy: case report and review of the literature. Can Med Assoc J.

